# *In Vitro* Evaluation of Antiprotozoal and Antiviral Activities of Extracts from Argentinean *Mikania* Species

**DOI:** 10.1100/2012/121253

**Published:** 2012-07-31

**Authors:** Laura C. Laurella, Fernanda M. Frank, Andrea Sarquiz, María R. Alonso, Gustavo Giberti, Lucia Cavallaro, Cesar A. Catalán, Silvia I. Cazorla, Emilio Malchiodi, Virginia S. Martino, Valeria P. Sülsen

**Affiliations:** ^1^Cátedra de Farmacognosia, Facultad de Farmacia y Bioquímica, Universidad de Buenos Aires, Junín 956 2°P, 1113, Buenos Aires, Argentina; ^2^Cátedra de Inmunología, IDEHU UBA-CONICET, Facultad de Farmacia y Bioquímica y Departamento de Microbiología, Facultad de Medicina, Universidad de Buenos Aires, Junín 956 4°P, 1113, Buenos Aires, Argentina; ^3^Cátedra de Virología, Facultad de Farmacia y Bioquímica, Universidad de Buenos Aires, Junín 956 4°P, 1113, Buenos Aires, Argentina; ^4^Instituto de Química y Metabolismo del Fármaco (IQUIMEFA), UBA-CONICET, Junín 956 4°P, 1113, Buenos Aires, Argentina; ^5^INQUINOA-CONICET y Instituto de Química Orgánica, Facultad de Bioquímica, Química y Farmacia, Universidad Nacional de Tucumán, Ayacucho 471, T4000INI San Miguel de Tucumán, Argentina

## Abstract

The aim of this study was to investigate the antiprotozoal and antiviral activities of four Argentinean *Mikania* species. The organic and aqueous extracts of *Mikania micrantha, M. parodii, M. periplocifolia,* and *M. cordifolia* were tested on *Trypanosoma cruzi* epimastigotes, *Leishmania braziliensis* promastigotes, and dengue virus type 2. The organic extract of *M. micrantha* was the most active against *T. cruzi* and *L. braziliensis* exhibiting a growth inhibition of 77.6 ± 4.5% and 84.9 ± 6.1%, respectively, at a concentration of 10 **μ**g/ml. The bioguided fractionation of *M. micrantha* organic extract led to the identification of two active fractions. The chromatographic profile and infrared analysis of these fractions revealed the presence of sesquiterpene lactones. None of the tested extracts were active against dengue virus type 2.

## 1. Introduction

Neglected tropical diseases (NTDs) are a group of infectious diseases that cause significant morbidity and mortality in the developing world. American Trypanosomiasis or Chagas' disease, leishmaniasis, and dengue are considered NTDs [1]. According to the World Health Organization (WHO), there are more than 1 billion people that suffer one or more NTD, mostly concentrated in countries of Africa, Asia, and Latin America, where life conditions are linked to poverty. As a consequence, the development of new or better drugs to fight these diseases is not a priority for the pharmaceutical industry. 

Chagas' disease, caused by the protozoan parasite *Trypanosoma cruzi*, affects 10 million people worldwide [2], and almost 12 million people are infected with *Leishmania* spp. [3]. Dengue is a viral infection caused by an RNA virus and it is estimated that 50–100 million cases occur annually while approximately half of the world's population is at risk [4].

Chagas' disease, leishmaniasis, and dengue are considered to be, among others, the most common tropical diseases in Argentina [5]. Between 1.5 and 2 million people are reported to be affected by Chagas' disease in this country [6]. American tegumentary leishmaniasis (ATL) is endemic in Northern Argentina where it is frequently associated with Chagas' disease [7] and its incidence, due in particular to *Leishmania braziliensis*, has increased during the last two decades [8]. On the other hand, a dengue epidemic outbreak in 2009 produced more than 25000 cases [9]. 

The drugs currently available to treat acute Chagas' disease infection are the nitroaromatic compounds, benznidazole and nifurtimox, both of which were discovered in the 70s'. They are effective only in the acute phase of the disease and have serious side effects [2]. The chemotherapy of leishmaniasis is based on pentavalent antimonials, amphotericin B, miltefosine and paromomycin which all have drawbacks [10]. In the case of dengue, however, currently, there are neither licensed vaccines nor any available drug to treat this viral infection [11]. In view of this situation, there is an urgent need to find new drugs to treat these NTDs. 

Natural products have played an important role in the drug discovery process, since they are generally small molecules with a wide chemical diversity and more “drug-likeness” than synthetic compounds, so that they are good candidates for lead drug development [12, 13].

In the last decades, the Asteraceae family has been extensively studied due to the great number and variety of active compounds isolated from species belonging to it. Among these, the genus *Mikania*, which comprises nearly 450 species [14], has been reported to contain some interesting chemical substances, mostly terpenoid compounds (sesquiterpene lactones and diterpenes) and flavonoids. These secondary metabolites are known to have important biological activities, including anti-infective properties [15–18]. There are no previous reports concerning the evaluation of *Mikania* spp. on dengue virus, though *M. micrantha* has been reported to be effective against respiratory viruses [19]. 

The aim of the present study, thus, was to determine the *in vitro* antiprotozoal and antiviral activities of four Argentinean *Mikania* species. Organic and aqueous extracts of *Mikania micrantha, M. periplocifolia*, *M. parodii,* and *M. cordifolia* were tested against *Trypanosoma cruzi*, *Leishmania braziliensis,* and dengue virus.

## 2. Materials and Methods

### 2.1. Plant Material

The aerial parts of *Mikania micrantha *Kunth (Asteraceae) were collected in Tucumán Province, Argentina in June 2009. Botanical identification was performed by Lic. A. Slanis and Dr. B. Juarez. A voucher specimen (LIL 609699) was deposited at the Herbarium of Instituto Miguel A. Lillo.

*Mikania parodii* Cabrera (Asteraceae) (BAF 713) and *Mikania cordifolia* (L. f.) Willd. (Asteraceae) (BAF 715) were collected in May 2009 and *Mikania periplocifolia* Hook. & Arn. (Asteraceae) (BAF 732) in November 2011, in all cases in Entre Ríos Province. The plant material was identified by one of the authors and voucher specimens were deposited at the Museo de Farmacobotánica, Facultad de Farmacia y Bioquímica, Universidad de Buenos Aires.

### 2.2. Microorganisms

*Trypanosoma cruzi* epimastigotes (RA strain) were grown in a biphasic medium and *Leishmania braziliensis* promastigotes (2903 strain) in liver infusion tryptose medium (LIT). Cultures were routinely maintained by weekly passages at 28°C and 26°C, respectively.

The replication of dengue virus type 2 (DENV-2) (16681 strain) was performed using Vero cells (ATCC CCL-81), baby hamster kidney (BHK-21) clon 15. 

### 2.3. Preparation of Plant Extracts

The aerial parts of *Mikania micrantha, M. periplocifolia, M. parodii, *and* M. cordifolia *(20 g each) were air dried and extracted with dichloromethane/methanol (1 : 1) (200 mL) at room temperature for 24 h and then vacuum filtered. The process was repeated twice and the extracts were combined and dried under vacuum. The marc was then extracted with water in the same conditions. Aqueous extracts were freeze-dried. 

### 2.4. Fractionation of *Mikania *micrantha** Organic Extract

The aerial parts of *M. micrantha* (400 g) were extracted with dichloromethane/methanol (1 : 1) as described above. The organic extract was subjected to open column chromatography on silica gel 60 and eluted successively with hexane, hexane: ethyl acetate (1 : 1), ethyl acetate and methanol yielding 48 fractions of 250 mL each. According to their thin-layer chromatography profile, these fractions were combined into 8 final fractions (1–8). These were subsequently tested for trypanocidal activity against *T. cruzi* epimastigotes. 

### 2.5. High Performance Liquid Chromatography Analysis (HPLC)

The HPLC analysis of fractions 3 and 4 was performed on a Varian Pro Star instrument equipped with a Rheodyne injection valve (20 *μ*l) and a photodiode array detector set at 210 nm. 

A reversed-phase column Phenomenex—C18 (2) Luna (250 mm × 4.6 mm·5 *μ*.) was used. Samples were eluted with a gradient of water (A) and acetonitrile (B) from 0% B to 75% B in 30 min and 75% B to 100% B in 2 min. The flow rate was 1.0 mL/min and the separation was done at room temperature. Chromatograms were recorded and processed using the Varian Star Chromatography Workstation version 6.x.

Fractions 3 and 4 were dissolved in methanol and water (90 : 10) to a final concentration of 5 mg/mL. 

Water employed to prepare the mobile phase was of ultrapure quality (Milliq). Acetonitrile (HPLC) J. T. Baker and methanol (HPLC) J. T. Baker were used.

### 2.6. Infrared Spectroscopic Analysis

The IR-spectra of the active fractions 3 and 4 were recorded on FT-IR spectrophotometer (Bruker IFS-25) in chloroform solution.

### 2.7. Trypanocidal and Leishmanicidal Activity Assay

Growth inhibition of *T. cruzi *epimastigotes and *L. braziliensis* promastigotes was evaluated using the previously described [^3^H] thymidine uptake assay [15]. Parasites were adjusted to a cell density of 1.5 × 10^6^/mL and cultured in the presence of each extract or fraction for 72 h. Benznidazole (5 to 20 *μ*M; Roche) and Amphotericin B (0.27–1.6 *μ*M, ICN) were used as positive controls. The percentage of inhibition was calculated as 100 − {[(cpm of treated parasites)/(cpm of untreated parasites)] × 100} [16]. Organic and aqueous extracts of *Mikania* species were tested on both parasites at concentrations of 100, 10 and 1 *μ*g/mL for 72 h. Extracts which showed an inhibition below 30% at a concentration of 10 *μ*g/mL were no further tested. Fractions 1–8 were assayed on *T. cruzi* at concentrations of 100 and 10 *μ*g/mL.

### 2.8. Antiviral Activity Assay

Vero cells were seeded in Minimal Essential Medium (MEM), supplemented with 10% fetal bovine serum (FBS, PAA) in microwell plates (96 wells) at a density of 2.2 × 10^4^ cells per well. After 24 h in a 5% CO_2_ incubator at 37°C, the cells were infected with DENV-2 in MEM supplemented with 2% FBS that induced an 80–90% cytopathic effect (CPE) on the sixth day postinoculation in absence of the drug. 

The cytotoxic concentration 50% (CC_50_), defined as the concentration that inhibits the proliferation of exponentially growing cells by 50%, was calculated for organic and aqueous extracts. 

Two-fold serial dilutions of organic (12–0.75 *μ*g/mL) and aqueous extracts (500–31.25 *μ*g/mL) of *Mikania *species were tested in quadruplicate. Mock-infected cells with and without extracts and infected cells without extract were included as controls. Ribavirin (100–1 *μ*g/mL; Sigma-Aldrich) was used as a positive control. The CPE was determined by the measurement of cell viability using the MTS/PMS method (CellTiter 96 Aqueous) (Promega, Madison, WI) as previously described [20]. All extracts were tested at concentrations below their CC_50_.

### 2.9. Statistical Analysis

The results are expressed as mean ± SEM. The level of statistical significance was determined submitting the data to one-way analysis of variance (ANOVA) using GraphPad Prism 3.0 software (GraphPad Software Inc., San Diego, CA). All data were referred to the control group. *P* values of <0.05 were considered significant.

## 3. Results

### 3.1. Trypanocidal Activity

The trypanocidal activity of organic and aqueous extracts of *Mikania* species was evaluated *in vitro* on *T. cruzi* epimastigotes. Results are presented in Table 1. 

The organic extracts of the four *Mikania* species were found to be active against *T*. *cruzi* epimastigotes with an inhibition above 85% at 100 *μ*g/mL. The organic extract of *M*. *micrantha* proved to be the most active of all tested species, showing an inhibition of 77.6 ± 4.5% and 14.2 ± 4.6%, when applied at concentrations of 10 and 1 *μ*g/mL, respectively. The aqueous extracts of the four *Mikania* species showed inhibitions ranged between 13 and 40% at 100 *μ*g/mL (Table 1).

According to these results, the organic extract of *M. micrantha* was selected for further fractionation.

### 3.2. Leishmanicidal Activity

The leishmanicidal effect of extracts of *Mikania* species was evaluated on *L. braziliensis* promastigotes. The results are shown in Table 2.

At a concentration of 100 *μ*g/mL, organic extracts of *M. micrantha* and *M. parodii* displayed leishmanicidal activity with growth inhibition rates above 85%. At the lowest concentration tested (1 *μ*g/mL), *M. micrantha* was the most active extract with an inhibition of 77.8 ± 1.1%.

Aqueous extracts displayed inhibition rates below 30% at a concentration of 10 *μ*g/mL.

### 3.3. Antiviral Activity

None of the organic and aqueous extracts from the four tested *Mikania* species was able to inhibit the replication of DENV-2. Approximately 30% reduction of the CPE effect was observed when infected cells were treated with 500 *μ*g/mL aqueous extracts.

### 3.4. Bioassay-Guided Fractionation of *Mikania *micrantha** Organic Extract

**Figure 1:**
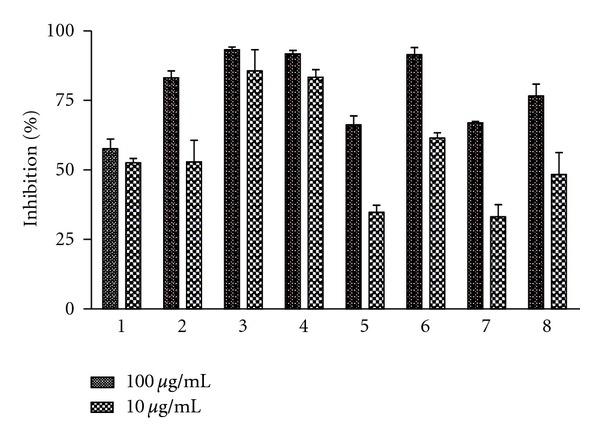
Trypanocidal activity of fractions 1–8 of *Mikania micrantha* on *T. cruzi* epimastigotes.

The fractionation of the organic extract of *M. micrantha* by column chromatography yielded eight final fractions (1–8), which were tested *in vitro* against *T. cruzi* epimastigotes. Fractions 3, 4, and 6, at a concentration of 100 *μ*g/mL, showed trypanocidal activity with percentages of growth inhibition of 93.2 ± 1.0%, 91.8 ± 1.2%, and 91.4 ± 2.6%, respectively (Figure 1).

**Figure 2:**
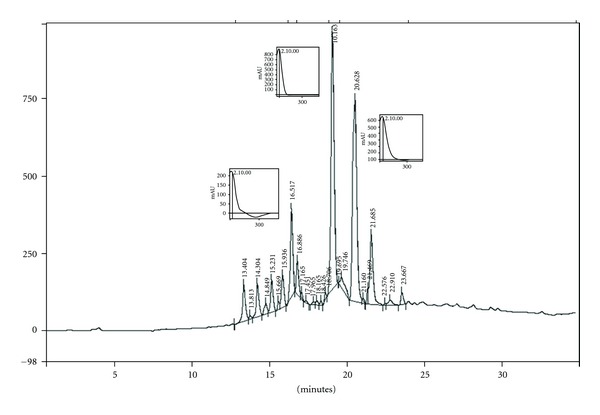
HPLC chromatographic profile of fraction 4 from *Mikania micrantha*.

At the lowest tested concentration (10 *μ*g/mL), fractions 3 and 4 were the most active against *T. cruzi* with inhibition rates of 85.7 ± 7.6% and 83.4 ± 2.8%, respectively (Figure 1). The analysis of the HPLC profile of these two fractions showed the presence of the same three major peaks with retention times of 16.5, 19.2, and 20.6 min and UV maximum at 219, 217, and 223 nm, respectively (Figure 2).The infrared spectroscopic analysis of these fractions showed the presence of bands between 1750–1790 cm^−1^, corresponding to *γ*-lactone carbonyl group (data not shown).

## 4. Discussion

In the present study, the antiprotozoal and antiviral effects of extracts of four Argentinean *Mikania* species against *Trypanosoma cruzi, Leishmania braziliensis,* and dengue virus type 2 were evaluated.

The organic extracts of *M. micrantha*, *M. periplocifolia*, *M. parodii,* and *M. cordifolia* showed significant *in vitro* antiprotozoal activity against *T. cruzi* epimastigotes and *L. braziliensis* promastigotes. The *M. micrantha* organic extract was the most active against the two protozoans. All aqueous extracts displayed moderate to low activity against *T. cruzi* and *L. braziliensis*. This is the first time that trypanocidal and leishmanicidal activities of *M. parodii* are reported.

In the case of antiviral activity, neither the organic nor the aqueous extracts were able to inhibit the replication of dengue virus type 2 under the described experimental conditions.

Previous reports on the chemical composition of species of the genus *Mikania* (Asteraceae) describe terpenoid compounds and flavonoids as the main constituents [21–24]. There are some references about the antiprotozoal and antiviral activities of *Mikania* spp. [19, 25, 26] and particularly, in the case of the four studied species, there are some reports of studies performed on different strains, stages, and parasite species than the ones used herein [27–29]. 

The bioguided fractionation of *M. micrantha* organic extract resulted in the identification of the most active fractions (3, 4) against *T. cruzi*. The chromatographic profile of these fractions revealed the presence of three major peaks with UV spectra that could be attributed to terpenoid compounds. Besides, in the infrared spectrum of these fractions, characteristic bands of sesquiterpene lactones could be observed. Thus, the active fractions 3 and 4 contain sesquiterpene lactones.

Terpenoids, mainly sesquiterpene lactones and diterpenes, are characteristic constituents of the genus *Mikania* and some of these metabolites have shown trypanocidal activity [24]. Thus, the trypanocidal activity of fractions 3 and 4 could be due to the presence of sesquiterpene lactones, since some of these compounds have been previously reported in *M. micrantha* [30–32].

These findings reveal the importance of the *Mikania* genus as a rich source of antiprotozoal molecules. Isolation and purification of the bioactive compounds from the active fractions of *M. micrantha* and bioassay-guided fractionation of the other active extracts are under way.

## Figures and Tables

**Table 1:** Trypanocidal activity of organic and aqueous extracts of *Mikania micrantha*, *M. periplocifolia*, *M. parodii,* and *M. cordifolia. *

Species	Extract	% of growth inhibition ± SEM		
		100 *μ*g/mL	10 *μ*g/mL	1 *μ*g/mL
*Mikania micrantha*	Organic	91.1 ± 3.7	77.6 ± 4.5	14.2 ± 4.6
	Aqueous	40.4 ± 1.4	23.2 ± 2.9	n.d.
*Mikania periplocifolia*	Organic	95.5 ± 0.4	56.7 ± 5.0	7.0 ± 4.2
	Aqueous	40.2 ± 2.5	25.2 ± 1.0	n.d.
*Mikania parodii*	Organic	94.9 ± 0.5	33.0 ± 1.3	2.3 ± 1.5
	Aqueous	30.2 ± 2.0	19.0 ± 1.8	n.d.
*Mikania cordifolia *	Organic	86.2 ± 1.8	10.5 ± 2.5	n.d.
	Aqueous	13.6 ± 2.8	12.2 ± 5.4	n.d.

Results are expressed as mean ± SEM. n.d.: not determined.

**Table 2:** Leishmanicidal activity of organic and aqueous extracts of *Mikania micrantha*, *M. periplocifolia*, *M. parodii,* and *M. cordifolia. *

Species	Extract	% of growth inhibition ± SEM		
		100 *μ*g/mL	10 *μ*g/mL	1 *μ*g/mL
*Mikania micrantha*	Organic	90.9 ± 0.8	84.9 ± 6.1	77.8 ± 1.1
	Aqueous	41.6 ± 4.1	29.9 ± 1.5	n.d.
*Mikania periplocifolia*	Organic	73.4 ± 5.3	69.2 ± 2.0	53.5 ± 4.3
	Aqueous	78.4 ± 7.2	11.4 ± 4.0	n.d.
*Mikania parodii*	Organic	87.3 ± 1.7	73.0 ± 0.6	58.7 ± 3.9
	Aqueous	48.7 ± 8.4	7.7 ± 1.5	n.d.
*Mikania cordifolia*	Organic	69.7 ± 6.6	55.7 ± 7.4	35.3 ± 7.5
	Aqueous	38.9 ± 3.5	5.0 ± 1.1	n.d.

Results are expressed as mean ± SEM. n.d.: not determined.
